# Influence of pH Cycling on Erosive Wear and Color Stability of High-Viscosity Glass Ionomer Cements

**DOI:** 10.3390/ma15030923

**Published:** 2022-01-25

**Authors:** Maja Zečević Čulina, Valentina Brzović Rajić, Ivan Šalinović, Eva Klarić, Luka Marković, Ana Ivanišević

**Affiliations:** School of Dental Medicine, University of Zagreb, Gundulićeva 5, 10000 Zagreb, Croatia; mzecevicc@sfzg.hr (M.Z.Č.); isalinovic@sfzg.hr (I.Š.); eklaric@sfzg.hr (E.K.); markovi.luka@gmail.com (L.M.); aivanisevic@sfzg.hr (A.I.)

**Keywords:** glass ionomer cement, erosion, mass change, colorimetry

## Abstract

The purpose of this in vitro study was to evaluate erosive wear and change in color of high-viscosity glass ionomer cements after pH cycling in two erosive media. There were 3 experimental groups with 22 samples each, (I) EQUIA Forte HT without coat, (II) Fuji IX and (III) Ketac Universal Aplicap. Each group was randomly divided into three subgroups (*n* = 6–8) further exposed to different environments, (1) distilled water, (2) green tea (pH 3.78) and (3) Aceto balsamico vinegar (pH 3.0). Mass and L* a* b* values were recorded before and after pH cycling. The samples in subgroups 2 and 3 were exposed to the acidic media two times a day for 10 min, over a period of 14 days. The differences among materials and erosive effects of the three media were tested using three-way analyses of variance with post hoc LSD test at the significance level *p* < 0.05. The effect of pH cycling in Aceto balsamico and green tea was degrading for all three materials. pH cycling in Aceto balsamico caused significantly higher erosive wear than pH cycling in Fuzetea and storage in distilled water, in all materials (*p* < 0.05). pH cycling in both acidic media and in the control group resulted in a significant change in L* a* and b* (*p* < 0.05). The L* value decreased significantly and the a* and b* values increased significantly (*p* < 0.05).

## 1. Introduction

Dental erosion is an irreversible loss of hard dental tissues caused by an acid dissolution of apatite crystals in enamel and dentin, without a direct association with bacterial plaque acids and caries, or other factors that might cause hard dental tissue loss, such as mechanical or traumatic factors [1]. Depending on the origin of the acid, erosions can be regarded as endogenous (gastroesophageal reflux disease (GERD), bulimia, regurgitation, rumination, etc.) or exogenous (dietary, occupational and drug-induced erosions). One of the most common exogenous causes of erosion is frequent and excessive intake of acidic foods and beverages, such as fruit juices, citric fruits, coffee, teas, salad dressings and alcohol [1,2,3,4]. The increasing prevalence of erosion in recent years has been associated with the increased consumption of soft drinks, but also with the increasing number of people following a vegetarian diet, which includes more frequent consumption of citrus fruits, acidic berries and vinegar [4,5,6]. Hartz et al. [4] recently reported that some salad dressings have an erosive potential higher than orange juice and the erosive effect was especially pronounced with pure balsamic-vinegar-based salad dressings. Besides affecting hard dental tissues, prolonged or periodical exposure to erosive media in the oral cavity can also affect the erosive wear of restorative materials, which can consequently jeopardize the success of restoration [7,8,9,10].

Glass ionomer cements (GIC) are two-component materials consisting of fluoroaluminosilicate glass and polyacrylic acid. The advantages of GICs include chemical binding to hard dental tissues, tolerance to the presence of moisture, a coefficient of thermal expansion similar to that of dentin, biocompatibility, bioactivity and easy manipulation, all of which make them widely used materials in contemporary dentistry [8,11]. The disadvantages of GICs, such as weaker physical and mechanical properties than dental resins, are, to a certain point, compensated by the development of recent materials based on GIC cements with high powder–liquid ratio (p/l), small reactive glass particles and nano-filled coats [11,12]. High-viscosity GICs are cements mixed with a higher p/l; therefore, they possess better physical and mechanical properties than conventional GICs [11,12]. Several such materials are available on the market, such as Ketac Universal Aplicap (ESPE, Neuss, Germany) and Fuji IX GP (GC, Tokyo, Japan). With the attempt to widen the indications for the use of high-viscosity GICs in the posterior region, microlaminated glass ionomer cements have been developed, where high-viscosity GICs were combined with a light-cured nano-filled coating [11]. Clinical evaluations of microlaminated GICs have proved them to be appropriate for long-term class I direct restorations and smaller class II direct restorations on posterior teeth [11]. Further modifications included the addition of highly reactive smaller particles to the powder to strengthen the material, which, together with the nano-filled light-cured coating, improved its mechanical properties [11,12]. An example of a glass-hybrid material is Equia Forte HT Fil (GC, Tokyo, Japan).

The results of previous in vitro and clinical studies have shown that erosive media affect direct restorations [7,8,9,10,13,14]. The disintegration of GICs in acidic environments occurs because H + ions from acid diffuse into the GIC material and displace metal cations from the matrix; the material dissolves and the components of its matrix, namely, calcium (or strontium) and aluminum cations, diffuse into the environment [10,13,15]. This further causes the release of metal cations from the glass particles, which also disintegrate [10]. In addition to pH, the degree of erosive action depends on the concentration of calcium ions and, to a lesser extent, on the concentration of other ions released by dissolving HAp crystals (phosphate, fluoride) or restorative material [4,5,6]. In other words, the saturation of the solution with ions released by erosion determines the direction of the chemical reaction (the erosion stops or progresses) at a certain pH and temperature [4,6]. The disintegration of restoration due to erosion leads to mass reduction, and rough surface of the material with microcavities and protruded, partly decomposed glass particles which favors staining and discoloration [10]. Therefore, it is very important to consider the resistance of a material to acidic load when assessing its suitability for restoration in certain clinical situations, such as class II open-sandwich restorations, especially in patients with a history of gastroesophageal reflux disease, eating disorders or consuming much acidic beverages and acidic foods [10,13,16].

The color stability of restorative materials can be assessed using the CIE (Fr. Commission Internationale de l’Eclairage) L* a* b* color space. The CIELAB system is obtained by converting the x, y and z values of the coordinate system into the variables L* a* and b*, enabling the quantification of the position of color in the three-dimensional color space [17]. This color system is highly applicable and is commonly used in scientific studies because the components of color (perceptual brightness and the four unique colors of human vision) are numerically described [17,18]. The discoloration of esthetic dental restorative materials may be caused by intrinsic or extrinsic factors [19]. Intrinsic factors imply changes in chemical structures including dissociation of metal-poliacrylate salts in the GIC matrix in acidic conditions, while extrinsic discoloration implies adsorption or absorption of extrinsic molecules causing discoloration where the increased roughness of the material’s surface, such as after exposure to acidic solutions, may favor adsorption or absorption of pigmented molecules [19]. Total color stability of a material can be estimated by calculating the total change in color ΔE considering all three values [20]. If the ΔE value is higher than 3.3, it is considered that the color stability of a restorative material is not sufficient [20]. Although the requirements for aesthetics in class I and smaller class II, where high-viscosity GIC fillings may be indicated, are not as great as in anterior teeth, it is important to inform the patient that the color change in these fillings may be significantly greater than in composite fillings. In the case of higher aesthetic expectations, a sandwich technique would be a choice rather than a complete GIC restoration.

The null hypotheses of this study are that exposing high-viscosity glass ionomer and glass-hybrid restorative materials to erosive pH cycling in commonly consumed vinegar and a soft drink would not cause changes in mass and color.

## 2. Materials and Methods

The following three groups of materials in encapsulated form were used in the experiment:

(1) EQUIA Forte HT Fil (GC, Tokyo, Japan), without coating (*n* = 22);
(2) Fuji IX (GC, Tokyo, Japan) (*n* = 22);
(3) Ketac Universal Aplicap (ESPE, Neuss, Germany) (*n* = 22).

The materials (Table 1) were mixed according to the manufacturer’s instructions and extruded into cylindrical Teflon molds with a diameter of 6 mm and a height of 2 mm. When extruding the material, the molds were placed on a glass slab and the material was extruded into each mold in slight excess and then another glass slab was placed over the molds to ensure good condensation of the material and the flat surface of the samples. After the initial setting, the samples were removed from the molds and placed in Petri dishes in deionized water at 37 °C for seven days to set completely in a NUVE ES 120 incubator (Nuve Sanayi Malzemeleri Imalat ve Ticaret AS, Ankara, Turkey).

After the complete setting, the mass of the samples was determined in grams to four decimal places using an NBL 254i (AE Adam GmbH, Felde, Germany) analytical scale and color readings were performed using an Easyshade Advance 4.0 (VITA Zahnfabrik, Bad Sackingen, Germany) spectrophotometer according to the CIELAB color space. Prior to the measurements, the spectrophotometer was calibrated according to the manufacturer’s instructions. All measurements were performed by one examiner (M.Z.Č.). Measurements were performed in triplicate and the arithmetic mean was used in further analyses.

The samples from each group were then randomly divided into the following three subgroups:

(1) Control subgroup—samples kept in distilled water at 37 °C for 14 days (*n* = 6);
(2) Samples exposed to Fuzetea (Green iced tea passion fruit no sugar; Coca-Cola Company, Atlanta, Georgia, USA; pH = 3.78; *n* = 8);
(3) Samples exposed to Aceto balsamico di Modena (Ponti S.p.A., Vignola, Italy; pH = 3.0; *n* = 8).

Samples from subgroups (2) and (3) were exposed to erosive media twice a day for 10 min, for 14 days. The pH of the erosive media was determined using a Multiparameter Benchtop Meter inoLab^®^ Multi 9630 IDS ionometer (WTW; Weilheim in Oberbayern, Germany). Acidity measurements were performed in triplicate. The mean pH of Aceto balsamico was 3.00 and that of Fuzetea 3.78. After each erosive pH cycle, the samples were washed and stored in distilled water at 37 °C until the next erosive pH cycle. After the 14-day period of pH cycling, mass and L* a* b* values were determined again. As previously, the measurements were performed in triplicate and the arithmetic means were calculated. The total change in color ΔE was calculated as previously described [20] using the following equation:

(1) $$∆E={\left(∆L^{2}+∆a^{2}+∆b^{2}\right)}^{\frac{1}{2}}$$

A post hoc power analysis was performed using G*Power software [21]. The post hoc power analysis was performed on the existing recordings. For the effect size obtained, 0.58, the used a significance level of 0.05 and the total sample size of 64, with 9 groups and 2 dependent measurements, the observed power was 0.898, so the sample size was considered adequate for this type of study. The differences among materials and erosive media were tested by methods of three-way analyses of variance with a post hoc LSD test at the significance level *p* < 0.05.

## 3. Results

### 3.1. Changes in Mass after pH Cycling

The observed mass reduction in all samples was significant (*p* < 0.01). Erosive cycling in Aceto balsamico led to a statistically significantly higher mass reduction than the green tea group (*p* < 0.015) and control group (*p* < 0.013). The difference between the control group and green tea group was not significant (*p* = 0.801) (Table 2). Although the erosive effect was less pronounced in Fuji IX, there was no statistically significant difference among the three materials in the same acidic environment (*p* = 0.506).

### 3.2. Changes in Color after pH Cycling

The changes in the L* a* and b* values within the CIELAB system were statistically significant for all samples considering the values before and after pH cycling (*p* < 0.01).

L* decreased significantly in all groups. The decrease in L* was significantly greater in Aceto balsamico than in the Fuzetea and control groups (*p* < 0.05), while the difference between Fuzetea and control group was not significant (*p* > 0.05). The decrease in L* did not significantly differ between Fuji IX and Ketac in the same environment, while it was significantly greater in Equia than in the other two groups (Table 3).

The values a* and b* increased significantly in each group (*p* < 0.01). The change in a* significantly differed among the three environments (*p* < 0.05). In Fuji and Equia, a* increased the most in Aceto, while, in Ketac, it increased the most in distilled water. In the same environment, the increase in a* was similar in Fuji IX and Equia (*p* > 0.05) and it was significantly different in Ketac compared with the other two materials (Table 4). The increase in b* was significant in all groups (*p* > 0.01). It differed significantly among materials (*p* > 0.01). In Fuji and Ketac, the increase in the b* value was greater in Aceto and Fuzetea, while, in Equia, it increased in distilled water the most. It differed significantly between the Fuzetea group and the control group (*p* > 0.05), while the difference between the Aceto and control groups, and Aceto and Fuzetea was not significant (*p* > 0.05) (Table 5).

## 4. Discussion

The results of the present study show that both erosive media, Aceto (pH = 3) and Fuzetea (pH = 3.78), had a degrading effect on high-viscosity GICs Fuji IX and Ketac Universal Aplicap and glass-hybrid Equia Forte HT Fil. Thus, both null hypotheses were rejected.

Exposing standardized GIC samples to acidic load and assessing the change in mass has been well established in previous studies as the method for testing the resistance of GICs to acid [15,16]. The conditioning period of one week at 37 °C allowed stabilization of the acid–base reaction of glass ionomer cements so that water uptake would not affect the mass recordings because it was shown that hygroscopic expansion of GICs occurred in the first 24 h [22,23].

The results showing mass reduction and changes in color components are in concordance with previous studies, which confirmed that acidic environments in the oral cavity led to the disintegration of GICs [2,10,13,15,24,25]. Furthermore, this research study showed that the mass of samples in all three materials decreased significantly more if exposed to Aceto balsamico vinegar than to Fuzetea and distilled water (*p* < 0.05). This is in concordance with Perera et al.’s report [16] and suggests that the availability of hydrogen ions (pH) is related to the extent of a material’s dissolution and disintegration. However, there was no statistically significant difference among the three groups of materials in certain pH environments (*p* > 0.05). This is not in concordance with Perera et al. [16], who reported that recent GIC formulations, such as glass-hybrid material Equia Forte Fil, showed higher resistance to erosion than high-viscosity GICs. Here, it should be emphasized that the samples of Equia Forte HT Fil glass-hybrid material were not protected by nano-filled coating in the present study. It was indeed reported that nano-filled coating significantly increased resistance to wear of Equia Forte HT Fil [12]. The composition and particle size of chemically set GIC materials was thus the variable influencing the resistance to erosion, while other factors, such as the application of protective varnishes and coats, were excluded. Since the differences among the materials under the same acidic conditions were not statistically significant, the results might imply that smaller particles, that the manufacturer claimed increased reactivity and chemical bonds between glass particle ions and polyacrylic acids, did not contribute to the resistance of the glass-hybrid material to the erosive action of Aceto and Fuzetea. On the contrary, high-viscosity Fuji IX eroded slightly less than Equia Forte HT Fil material. However, our results are in line with a recent report of Rai et al. [26] who exposed different restorative materials to acidic beverages (including resin-modified GICs, high-viscosity Fuji IX GICs and nano-GICs) and noticed maximum surface microhardness reduction in nano-GICs. It might be that the cations diffuse easier from small reactive filler particles than form the larger ones, causing faster cement disintegration. This assumption is supported by the dissolution of GICs as described by Wilson and Kuhn [27] where diffusion is a critical point before the actual corrosion of the cement.

The results of our study show that the L* a* and b* values changed significantly in all groups (*p* < 0.05). Sidhu et al. [18], in their study, also showed that GICs lacked color stability over time. In addition, Inokosi et al. [17] found that the opacity of GICs decreases and the material gets darker over time. This color instability can be explained by the hydrolysis and surface degradation of the material, especially in glass-hybrid material Equia Forte containing nanoparticles, which exhibited a relatively bigger change in L* and b* in distilled water than in high-viscosity materials. This is probably due to the surface erosion and hydrolysis of the glass-hybrid material without a protective coating in distilled water or an aqueous solution [28]. Furthermore, it was reported that the color stability of high-viscosity GICs can be negatively affected by the consumed beverages, especially coffee and cola [29]. The staining due to pigments adsorption and absorption is enhanced by the acidity of beverages, leaving the surface of the materials rough [10,26]. In the present study, the discoloration after continuous exposure to Aceto was more pronounced than in Fuzetea and deionized water in Fuji IX and Equia, but not in Ketac. This is in concordance with the finding that the mass reduction in Ketac groups, in both Fuzetea and Aceto, was not significant (Table 2). The deterioration of the Ketac material surface was probably less pronounced, making the material less prone to discoloration.

All the materials used in this study were chemical-cured materials belonging to high-viscosity GICs and glass-hybrid materials. The observed similar behavior of the materials when it comes to color stability and resistance to acidic challenge calls into question the tendency to classify Equia Forte as a separate restorative concept outside of the high-viscosity GIC group [11,12]. Indeed, the total ΔE in the present study of all materials in all conditions, including distilled water, was not acceptable (Table 6). Acetic acid is the component of Aceto balsamico and to find that L* a* and b* changed significantly in all the tested materials was not surprising, since it was reported that the color change in chemically cured GICs in a 0.1 mole acetic acid solution was significant [30]. The limitation of this study is that it did not consider possible effects of the erosion-modifying buffering capacity of saliva and salivary calcium concentration [4,6]. Furthermore, the effect of dietary combinations on erosion was not considered; for instance, a calcium-rich diet can significantly reduce the erosive effect of the acidic alimentary products consumed. pH alone does not determine the erosive potential and it was indeed shown that some highly acidic alimentary products, such as salad dressings with high calcium content, caused significantly lower erosive wear of enamel than less acidic salad dressings containing less calcium [4]. Furthermore, the effect of the mechanical loading and aging of the material was not considered. Therefore, the results should be observed considering the experimental conditions. Further in vitro and in vivo studies on the acid resistance of GIC-based restorative materials are required.

## 5. Conclusions

Within the limitations of this study, we can conclude that the erosive effect of pH cycling with Aceto balsamico (pH = 3.00) was significantly higher than the erosive effect of Fuzetea (pH = 3.78) for the EQUIA Forte HT Fil, Fuji IX and Ketac Universal Aplicap materials. The L* a* and b* values changed significantly in all experimental conditions and in all materials. The L* value decreased significantly and the a* and b* values increased significantly. The total ΔE was very high and was between 8.21 and 15.71 for the three GIC materials tested, which is clinically insufficient for the restorations where a high degree of esthetics is required [17].

## Figures and Tables

**Table 1:** Composition of high-viscosity GIC materials used in the study according to manufacturers.

Material	Type of Material	Manufacturer	Composition—Powder	Composition—Liquid
Fuji IX GP FAST	Conventional high-viscosity glass ionomer	GC, Tokyo, Japan	90–100% fluoroaluminosilicate glass, 5–10% polyacrylic acid	30–40% polyacrylic acid, polycarboxylic acid, 40% distilled water
Ketac Universal Aplicap	Conventional high-viscosity glass ionomer	3M ESPE, Neuss, Germany	oxide glass	copolymer of acrylic acid—maleic acid, tartaric acid, water
EQUIA Forte HT Fil	Glass-hybrid restorative material	GC, Tokyo, Japan	95% strontium fluoroaluminosilicate glass, 5% polyacrylic acid	40% aqueous polyacrylic acid

**Table 2:** The analysis of mass reduction in relation to the erosive media. Aceto balsamico led to a significantly higher mass reduction in all materials compared to Fuzetea and control (distilled water). The differences between the materials were not significant in the same environment.

	Mean ± Std dev	Before (mg)	After (mg)	Difference (µg)
Fuji IX	Fuzetea (*N* = 8)	0.1654 ± 0.0145	0.1649 ± 0.0149	−0.5958+/−0.7533 **
	Aceto balsamico (*N* = 8)	0.1596 ± 0.0112	0.1588 ± 0.0114	−0.7667+/−0.5164 **
	Control (*N* = 6)	0.1521 ± 0.0097	0.1521 ± 0.0096	−0.0389+/−0.1104
Ketac	Fuzetea (*N* = 8)	0.1574 ± 0.0080	0.1573 ± 0.0078	−0.1167+/−0.3314
	Aceto balsamico (*N* = 8)	0.1555 ± 0.0121	0.1524 ± 0.0140	−3.0792+/−7.8892
	Control (*N* = 6)	0.1545 ± 0.0059	0.1542 ± 0.0059	−0.2111+/−0.1708 *
Equia	Fuzetea (*N* = 8)	0.1472 ± 0.0112	0.1467 ± 0.0111	−0.4833+/−1.0673
	Aceto balsamico (*N* = 8)	0.1549 ± 0.0067	0.1506 ± 0.0056	−4.2875+/−4.3092 *
	Control (*N* = 6)	0.156 ± 0.00980	0.1558 ± 0.0098	−0.1889+/−0.1656 *
Total	All samples (*N* = 66)	0.156 ± 0.01090	0.1548 ± 0.0112	−1.1707+/−3.3352 **
Total	Fuzetea ^2^ (*N* = 24)	0.1567 ± 0.0134	0.1563 ± 0.0135	−0.3986+/−0.7723 ^2^ *
	Aceto balsamico ^1^ (*N* = 24)	0.1566 ± 0.0101	0.1539 ± 0.0110	−2.7111+/−5.1867 ^1^ *
	Control ^2^ (*N* = 18)	0.1542 ± 0.0083	0.1541 ± 0.0082	−0.1463+/−0.1626 ^2^ **
Total	Fuji IX ^1^ (*N* = 22)	0.1597 ± 0.0128	0.1592 ± 0.0129	−0.5061+/−0.6101 ^1^ **
	Ketac ^1^ (*N* = 22)	0.1559 ± 0.0090	0.1547 ± 0.0099	−1.2197+/−4.7813 ^1^
	Equia ^1^ (*N* = 22)	0.1524 ± 0.0098	0.1506 ± 0.0094	−1.7864+/−3.2148 ^1^ *

The differences marked by * were significant at *p* < 0.05 and the differences market by ** were significant at *p* < 0.01. The differences among changes in different materials marked by the same number (1, 2) were not significant and the differences marked by different numbers were statistically significant at *p* < 0.05.

**Table 3:** The analysis of L* showed that it reduced significantly after pH cycling in each group. Multiple comparisons based on observed means showed that the decrease in L* in Aceto balsamico was significantly different than in the Fuzetea and control groups. The decrease was significantly higher in Equia than in the other two groups.

	Mean ± Std dev	Before	After	ΔL
Fuji IX	Fuzetea (*N* = 8)	70.63+/−3.22	60.14+/−3.72	−10.49+/−6.12 **
	Aceto balsamico (*N* = 8)	73.05+/−0.91	60.10+/−2.55	−12.95+/−2.73 **
	Control (*N* = 6)	72.72+/−2.02	64.20+/−1.86	−8.52+/−2.45 **
Ketac	Fuzetea (*N* = 8)	74.04+/−0.42	66.19+/−0.99	−7.85+/−1.19 **
	Aceto balsamico (*N* = 8)	74.99+/−0.63	63.44+/−2.00	−11.55+/−2.22 **
	Control (*N* = 6)	74.15+/−1.19	66.82+/−0.83	−7.33+/−1.27 **
Equia	Fuzetea (*N* = 8)	78.11+/−1.55	63.93+/−3.36	−14.19+/−4.16 **
	Aceto balsamico (*N* = 8)	77.29+/−1.66	58.65+/−4.17	−18.64+/−5.26 **
	Control (*N* = 6)	76.65+/−3.10	64.77+/−2.60	−11.88+/−3.74 **
Total	All samples (*N* = 66)	74.63+/−2.90	62.94+/−3.76	−11.69+/−4.86 **
Total	Fuzetea ^2^ (*N* = 24)	74.26+/−3.7	63.42+/−3.8	−10.84+/−4.91 ^2^
	Aceto balsamico ^1^ (*N* = 24)	75.11+/−2.08	60.73+/−3.56	−14.38+/−4.69 ^1^
	Control ^2^ (*N* = 18)	74.51+/−2.69	65.26+/−2.13	−9.24+/−3.21 ^2^
Total	Fuji IX ^2^ (*N* = 22)	72.08+/−2.45	61.23+/−3.32	−10.85+/−4.44 ** ^2^
	Ketac ^2^ (*N* = 22)	74.41+/−0.85	65.36+/−2.02	−9.05+/−2.50 ** ^2^
	Equia ^1^ (*N* = 22)	77.41+/−2.09	62.24+/−4.36	−15.18+/−5.14 ** ^1^

The differences market by ** were significant at *p* < 0.01. The differences among changes in different materials and conditions marked by the same number (1, 2) were not significant and the differences marked by different numbers were statistically significant at *p* < 0.05.

**Table 4:** The analyses of a* showed that it increased significantly after pH cycling in each group. Multiple comparisons showed that the increase in a* in different environments was significantly different and was the highest in Aceto balsamico. The increase was significantly higher in Ketac than in Equia and Fuji IX, and it did not differ significantly in Equia and Fuji IX.

	Mean ± Std dev	Before	After	Δa
Fuji IX	Fuzetea (*N* = 8)	6.28+/−0.49	9.63+/−0.61	3.35+/−0.73 **
	Aceto balsamico (*N* = 8)	6.23+/−0.20	10.00+/−0.65	3.78+/−0.64 **
	Control (*N* = 6)	6.47+/−0.69	8.32+/−0.26	1.85+/−0.67 **
Ketac	Fuzetea (*N* = 8)	8.20+/−0.25	11.14+/−0.76	2.94+/−0.88 **
	Aceto balsamico (*N* = 8)	7.80+/−0.53	11.55+/−0.81	3.75+/−0.55 **
	Control (*N* = 6)	8.10+/−0.54	10.58+/−0.43	2.48+/−0.41 **
Equia	Fuzetea (*N* = 8)	6.60+/−0.32	8.94+/−0.6	2.34+/−0.57 **
	Aceto balsamico (*N* = 8)	6.76+/−0.35	10.15+/−0.64	3.39+/−0.65 **
	Control (*N* = 6)	6.63+/−0.49	8.75+/−0.59	2.12+/−0.50 **
Total	All samples (*N* = 66)	7.00+/−0.86	9.96+/−1.18	2.95+/−0.90 **
Total	Fuzetea ^2^ (*N* = 24)	7.03+/−0.93	9.90+/−1.13	2.88+/−0.82 ^2^ **
	Aceto balsamico ^1^ (*N* = 24)	6.93+/−0.76	10.57+/−0.98	3.64+/−0.61 ^1^ **
	Control ^3^ (*N* = 18)	7.07+/−0.93	9.22+/−1.09	2.15+/−0.57 ^3^ **
Total	Fuji IX ^2^ (*N* = 22)	6.31+/−0.46	9.40+/−0.88	3.10+/−1.03 ^2^ **
	Ketac ^1^ (*N* = 22)	8.03+/−0.47	11.14+/−0.78	3.11+/−0.82 ^1^ **
	Equia ^2^ (*N* = 22)	6.67+/−0.37	9.33+/−0.87	2.66+/−0.79 ^2^ **

The differences market by ** were significant at *p* < 0.01. The differences among changes in different materials and conditions marked by the same number (1, 2, 3) were not significant and the differences marked by different numbers were statistically significant at *p* < 0.05.

**Table 5:** The analyses of b* showed that it increased significantly after pH cycling in each group. The multiple comparison analysis showed that the increase in b* was significantly higher after pH cycling in Fuzetea than in distilled water, while the difference between Aceto balsamico and Fuzetea, and Aceto balsamico and control was not significantly different. The change in b* was significantly different in different materials.

	Mean ± Std dev	Before	After	Δb
Fuji IX	Fuzetea (*N* = 8)	35.26+/−1.5	40.95+/−1.57	5.69+/−2.28 **
	Aceto balsamico (*N* = 8)	35.89+/−0.61	40.90+/−0.93	5.01+/−1.06 **
	Control (*N* = 6)	36.53+/−1.27	39.33+/−0.67	2.80+/−1.51 **
Ketac	Fuzetea (*N* = 8)	46.34+/−0.61	49.34+/−1.45	3.00+/−1.72 **
	Aceto balsamico (*N* = 8)	45.65+/−0.99	48.48+/−1.24	2.83+/−1.38 **
	Control (*N* = 6)	46.15+/−0.94	48.88+/−0.83	2.73+/−0.68 **
Equia	Fuzetea (*N* = 8)	30.10+/−0.72	36.43+/−1.51	6.33+/−1.37 **
	Aceto balsamico (*N* = 8)	30.40+/−0.79	35.65+/−0.80	5.25+/−0.73 **
	Control (*N* = 6)	29.88+/−1.06	33.87+/−0.80	3.98+/−1.46 **
Total	All samples (*N* = 66)	37.34+/−6.69	41.61+/−5.75	4.27+/−1.92 **
Total	Fuzetea ^1^ (*N* = 24)	35.84+/−1.23	40.49+/−1.32	4.65+/−2.02 ** ^1^
	Aceto balsamico ^2^ (*N* = 24)	46.04+/−0.87	48.9+/−1.23	2.86+/−1.32 ** ^2^
	Control ^2^ (*N* = 18)	30.15+/−0.83	35.45+/−1.49	5.30+/−1.48 ** ^2^
Total	Fuji IX ^3^ (*N* = 22)	37.23+/−6.99	42.24+/−5.65	5.00+/−2.28 ** ^3^
	Ketac ^2^ (*N* = 22)	37.31+/−6.49	41.68+/−5.46	4.36+/−1.52 ** ^2^
	Equia ^1^ (*N* = 22)	37.52+/−6.95	40.69+/−6.43	3.17+/−1.33 ** ^1^

The differences marked by ** were significant at *p* < 0.01. The differences among changes in different materials and conditions marked by the same number (1, 2, 3) were not significant and the differences marked by different numbers were statistically significant at *p* < 0.05.

**Table 6:** Considering L* a* and b* values within the CIELAB system, the total color change was calculated for all three materials in all environments.

		Fuji IX			Ketac			Equia	
	Aceto	Fuzetea	Control	Aceto	Fuzetea	Control	Aceto	Fuzetea	Aceto
ΔL	−12.95	−10.48	−8.52	−7.85	−10.85	−7.33	−9.05	−7.33	−14.19
Δa	3.78	3.35	1.85	2.93	3.095	3.75	3.12	2.48	2.33
Δb	5.01	5.69	2.8	3	4.65	2.83	2.86	2.73	6.33
ΔE	14.39	11.16	9.16	8.89	12.2	8.71	9.99	8.21	15.71

## Data Availability

The data that support the findings of this study are available from the first author and corresponding author upon request.
